# Green Synthesis of Silver Nanoparticles Mediated by *Punica granatum* Peel Waste: An Effective Additive for Natural Rubber Latex Nanofibers Enhancement

**DOI:** 10.3390/polym16111531

**Published:** 2024-05-29

**Authors:** Talia S. Echegaray-Ugarte, Andrea L. Cespedes-Loayza, Jacqueline L. Cruz-Loayza, Luis A. Huayapa-Yucra, Isemar Cruz, Júlio Cesar de Carvalho, Luis Daniel Goyzueta-Mamani

**Affiliations:** 1 Sustainable Innovative Biomaterials Department, Le Qara Research Center, Arequipa 04000, Peru; t.echegaray@leqara.com (T.S.E.-U.); acespedesl@outlook.com.pe (A.L.C.-L.); j.cruz@leqara.com (J.L.C.-L.); l.huayapa@leqara.com (L.A.H.-Y.); i.cruz@leqara.com (I.C.); 2 Bioprocess Engineering and Biotechnology Department, Federal University of Paraná—Polytechnic Center, Curitiba 81531-980, Brazil; jccarvalho@ufpr.br; 3 Vicerrectorado de Investigación, Universidad Católica de Santa María, Urb. San José s/n-Umacollo, Arequipa 04000, Peru

**Keywords:** *Punica granatum*, green synthesis, silver nanoparticles, natural rubber latex, antibacterial activity

## Abstract

Pomegranate waste poses an environmental challenge in Arequipa. Simultaneously, interest in sustainable materials like natural rubber latex (NRL) is growing, with Peruvian communities offering a promising source. This study explores the green synthesis of silver nanoparticles (AgNPs) using pomegranate peel extract and their incorporation into NRL nanofibers for enhanced functionalities. An eco-friendly process utilized silver nitrate and pomegranate peel extract as a reducing and capping agent to synthesize AgNPs. The resulting AgNPs and NRL/AgNPs nanofibers were characterized using imaging and spectroscopic techniques such as UV-vis, TGA, FTIR, XRD, Raman, SEM, and DLS. Green-synthesized AgNPs were spherical and crystalline, with an average diameter of 59 nm. They showed activity against *K. pneumoniae*, *E. coli*, *B. cereus*, and *S. aureus* (IC50: 51.32, 4.87, 27.72, and 69.72 µg/mL, respectively). NRL and NRL/AgNPs nanofibers (300–373 nm diameter) were successfully fabricated. The composite nanofibers exhibited antibacterial activity against *K. pneumoniae* and *B. cereus*. This study presents a sustainable approach by utilizing pomegranate waste for AgNP synthesis and NRL sourced from Peruvian communities. Integrating AgNPs into NRL nanofibers produced composites with antimicrobial properties. This work has potential applications in smart textiles, biomedical textiles, and filtration materials where sustainability and antimicrobial functionality are crucial.

## 1. Introduction

Nanotechnology is a vibrant, expanding field, producing a plethora of unique nanomaterials for applications in biotechnology and nanomedicine. The synthesis of these nanomaterials using biological methods offers a simple, non-toxic, and reliable strategy, presenting a more eco-friendly alternative to costly and hazardous chemical procedures [1].

These biologically synthesized nanomaterials exhibit superior biocompatibility compared to their chemically synthesized counterparts. They are devoid of harmful impurities typically produced during chemical synthesis. Moreover, additional stabilizing agents are unnecessary for biosynthesis as the organisms or their metabolic products can act as capping and stabilizing agents [2]. This biological approach, particularly the synthesis of metal nanoparticles like Ag, Cu, Au, and others using plant extracts or waste, is gaining traction due to its simplicity, safety, scalability, and the potential for recycling agro-industrial waste [3].

Silver nanoparticles (AgNPs) stand out among metal nanoparticles due to their unique physical and chemical properties, including conductivity, chemical stability, and catalytic and antibacterial activity [1]. Their antimicrobial action arises from multiple mechanisms. Firstly, AgNPs can adhere to bacterial cell walls and membranes, causing structural damage. They also penetrate cells, disrupting vital intracellular components like mitochondria, ribosomes, and biomolecules (DNA, proteins, lipids). AgNPs further induce oxidative stress by generating reactive oxygen species (ROS) and free radicals, which are toxic to cells. Additionally, it can disrupt signal transduction pathways essential for microbial function. Importantly, AgNPs also release silver ions (Ag^+^), which denature proteins by interacting with sulfhydryl groups. Finally, AgNPs can modulate the host immune system, triggering an inflammatory response that assists in combating infections [4,5].

Interestingly, *Punica granatum*, a crop extensively cultivated in the Arequipa region, generates significant waste. Peru’s pomegranate production, as reported by the Peruvian Agricultural Ministry [6], has substantially increased, from 928.2 tons in 2000 to 46,382.9 tons in 2018. This represents an average annual growth rate of 24.3%, with the harvested area expanding from 90 hectares in 2000 to approximately 2800 hectares [6]. Talekar et al., 2018 [7] reported that processing one ton of fresh pomegranates results in 500–550 kg of waste peels as a byproduct. For comparison, California generated around 118,000 tons of pomegranate peel and seed waste in 2018, from a total production of over 218,000 tons of pomegranates [8]. The inedible parts of fruits and trees, such as peel, seeds, blossoms, bark, buds, and leaves, are often discarded as waste. However, surprisingly, these parts contain more specific biologically active components than the edible ones. They are rich in polyphenolic compounds such as punicalagin isomers, ellagic acid derivatives, and anthocyanins. These compounds not only serve as stabilizing and capping agents during the conversion of metallic salts to metallic nanoparticles but also offer significant health benefits [9,10]. The pomegranate peel, which makes up approximately 50 percent of the fruit’s weight, possesses significant free radical scavenging, antibacterial, antiatherogenic, and antimutagenic effects. Thus, pomegranate residues, especially the peel, represent an attractive and underutilized bioresource for synthesizing bioactive nanoparticles with potential medicinal applications [11].

Electrospinning can incorporate metallic nanoparticles derived from pomegranate residue extracts into various matrices. This process involves creating a charged jet stream from a polymer solution, which is then stretched, dried, and solidified using electrostatic forces to produce a fiber [12]. While initial studies on electrospun biodegradable polymers primarily focused on synthetic materials like polycaprolactone, polylactic acid, and polyglycolic acid [13], recent research is shifting towards using biomaterials and creating eco-friendly composites from naturally sourced raw materials [14].

One such revolutionary biomaterial is the natural rubber latex (NRL) derived from the rubber tree, categorized among biopolymers as elastomers [15]. The rubber tree (*Shiringa tree*), scientifically known as *Hevea brasiliensis*, is a species native to the Amazon rainforest and a significant latex source. The commercial activity related to Shiringa is forest-friendly, involving latex collection from tree cross-sections without deforestation [16]. This latex is an economically advantageous and sustainable resource for the Peruvian Amazon community [17]. The NRL is a renewable polymer boasting unique properties such as electrical insulation, elasticity, flexibility, resistance to abrasion and corrosion, and accessible surface adhesion. These attributes make it a valuable raw material across various industries, leading to a rising demand for sustainably produced natural rubber [18].

This research aims to synthesize AgNPs biogenically from the extract of Punica granatum peel and integrate them into an NRL matrix. By harnessing the reducing and stabilizing properties of pomegranate peel extract, a potentially valuable waste of local agriculture, this work offers an eco-friendly alternative to nanoparticle synthesis while supporting the conscious production of local communities. Moreover, integrating these nanoparticles into NRL could create biomaterials with unique antibacterial properties, addressing a significant need in various applications. This work holds the potential to improve the field by demonstrating a sustainable approach for creating functionalized biomaterials, expanding the range of applications for both NRL and biogenically synthesized nanoparticles.

## 2. Materials and Methods

### 2.1. Preparation of Pomegranate Extract and Characterization

Pomegranates (*Punica granatum*) were donated by Fundo América SAC (Arequipa, Peru). These fruits were wastes and byproducts that did not meet the quality criteria for exportation. The donated pomegranates were thoroughly washed with deionized water to eliminate surface contaminants. The peels were meticulously separated from the pulp using a sharp knife. The peels were chopped into small squares with dimensions of approximately 1–2 cm^2^ to facilitate efficient drying. Finally, the chopped peels were evenly distributed on a clean tray and air-dried at an elevated temperature of 40 °C until they reached a state of complete dryness.

Hydroalcoholic extracts were obtained by mixing an ethanol solution (1:4 water/ethanol ratio) in a 1:5 ratio of dry peel to final volume. The mixture was stirred at room temperature at 120 rpm for 72 h, filtered, and centrifuged at 5000 rpm for 5 min to avoid interference or detritus. The pomegranate fruits were donated by the company Fundo América SAC (Arequipa, Peru).

Further analyses were performed to evaluate the properties of the hydroalcoholic extract obtained from pomegranate peel. Total sugars were determined spectrophotometrically using the 3,5-dinitrosalicylic acid (DNS) method using a standard glucose solution at 540 nm (DLAB^®^, SP-V1100, Beijing, China) [19]. The antioxidant capacity was measured using the 2,2-diphenyl-1-picrylhydrazyl (DPPH) method, with ascorbic acid solution as the standard at a wavelength of 517 nm [20]. Spectrophotometric analysis at 750 nm determined the total phenols following the Folin–Ciocalteu method using a standard gallic acid solution [21].

The total flavonoids were quantified using the method by Zhishen et al. (1999) [22], which involved a spectrophotometric analysis at 510 nm and a catechin solution as a reference standard. The reducing capacity was determined using the Ferric Chloride Test with an ascorbic acid solution reference at 700 nm [23]. The pomegranate peel extract was centrifuged at 2500 rpm for 5 min before sample collection for each test.

This characterization was performed in order to evaluate its potential as a reducing, stabilizing, and capping agent due to the chemical characteristics present in pomegranate peel.

### 2.2. Biogenic Synthesis of AgNPs and Optimization Process

AgNPs were synthesized using 10 mL of pomegranate peel hydroalcoholic extract as a reducing and capping agent, mixed with a 50 mL silver nitrate solution (10 mM). The mixture was placed in 100 mL beakers and protected from light during synthesis. A Box–Behnken experimental design (DBB) with 3 levels was carried out to improve the diameter of AgNPs. This evaluation was executed using the STATISTICA^®^ Version 10 software (Tulsa, OK, USA) with four independent variables: temperature, agitation, AgNO_3_ concentration, and reaction time (Table 1) [24]. pH was adjusted at 8. After the reaction time, the samples were centrifugated at 8500 rpm for 7 min, washed twice with distilled water, and resuspended in 10 mL of pure water. The samples were then stored at room temperature in a dark setting to avoid oxidation and deterioration.

### 2.3. Characterization of AgNPs

Various methods were employed to identify and characterize the synthesized AgNPs:

- Fourier transform infrared spectroscopy (FTIR) was used to analyze the functional groups of AgNPs by examining their chemical structure in the FTIR (FTIR-Bruker Alpha II, Karlsruhe, Germany) spectrum between 4000 and 500 cm⁻^1^.
- UV–visible spectroscopy (Genesys 10S UV-vis) was utilized to detect the presence of AgNPs. The wavelength range used to measure absorbance ranged from 300 to 800 nm. UV-vis spectroscopy comprises both the ultraviolet and visible spectra.
- Scanning electron microscopy (SEM) and Energy-Dispersive X-ray Spectroscopy (EDS) were used to analyze the surface morphology of AgNPs. Scanning electron microscopy (Thermo Scientific Scios 2, Waltham, MA, USA) was used to capture images and EDS spectra at 20 kV at a working distance of 20 mm.
- X-ray diffraction (XRD) was employed to analyze silver nanoparticles’ chemical composition and crystal structure. The equipment (XRD-Miniflex 600 W, Tokyo, Japan) was operated using a current of 15 mA and a voltage of 40 kV, together with a monochromator. Sweeping was conducted in increments of 0.02° at a speed of 2° per minute. A range of 20° to 80° was covered throughout the sweep. Glass sample holders were used. The average size of the silver nanoparticles was calculated using the Debye–Scherrer equation. The equation is as follows:

D = kλβcosθ

where D = particle diameter size; k = shape factor (0.94); λ = X-ray wavelength (λ = 0.1541 nm); β = full width at half maximum (FWHM) in radians and θ = diffraction angle.

- The dynamic light scattering (DLS) and Zeta potential model nano ZS-90 IESMAT brand and Zetasizer were utilized to analyze the size distribution of particles by sensing dynamic fluctuations in light scattering intensity due to Brownian motion. The measurement included the mean hydrodynamic diameter of the particles, the maximum values in the hydrodynamic diameter distribution, and the polydispersity index (PdI) that indicates the width of the particle size range. The PdI scale ranges from 0 to 1, where 0 represents monodisperse, and values greater than 1 indicate polydisperse.

### 2.4. Antimicrobial Capacity of AgNPs

Silver nanoparticles were tested for their antibacterial activity on pathogenic strains of Gram-negative *E. coli* (ATCC 10536), *K. pneumoniae* (ATCC 70068), and Gram-positive *S. aureus* (ATCC 27853) and *Bacillus cereus* (ATCC 99815) using the minimum inhibitory concentration (MIC) method. Dilutions were prepared at six distinct concentrations: 70, 35, 17.5, 8.7, 4.3, and 2.1 µg/mL. The inoculum was prepared at 1 × 10^8^ CFU/mL (0.50 ± 0.033 on the McFarland scale). The microorganisms were resuspended and measured for turbidity using a densitometer (BIOSAN model DEN-1B). Chloramphenicol was used as a positive control at 2 mg/mL concentration. A 96-well microplate was filled with nutritional broth (80 µL), bacterial suspension (20 µL), and AgNPs in suspension (100 µL). The microplates were kept at 37 °C for 24 h. Subsequently, resazurin (0.1%) was added and incubated for another 2 h. The absorbances were measured using a spectrophotometer at a wavelength of 630 nm (MINDRAY–MR-96A) [25].

### 2.5. Photocatalytic Activity of AgNPs

An amount of 2.5 mg of AgNPs was added to 25 mL of an aqueous methylene blue (MB) solution with a concentration of 10 mg/L to study the photocatalytic activity. Two different pH levels, 3 and 8, were also used to evaluate the efficacy. The resulting solution was then shaken and exposed to sunlight for 72 h. Two-milliliter samples were collected at time intervals. The supernatant solution was analyzed using a UV-vis spectrophotometer (Genesys 10S UV-vis) at 640 nm (*λ*max). A positive control of MB solution containing 10 mg/L without AgNPs was used, and NaHBr_4_ was used as the negative control. According to the Beer–Lambert law, the concentration of the colorant is directly proportional to the absorption, so its degradation rate can be calculated using the equation:

$$\%\;Degradation=\frac{Co-Cf}{Co}\times 100$$

where Co is the absorption of a blank solution, and Cf is the absorption of the sample after t minutes of exposure to visible light.

### 2.6. Electrospinning of NRL Decorated with AgNPs

Natural rubber latex (NRL) was a crucial component donated by Empresa Comunal Jebe Natural del MAP Tahuamanu (ECOMUSA) and Ecotahu SAC for this investigation. To ensure the NRL’s stability during transport and prevent premature coagulation, it was received as a liquid mixture containing urea and stored in flasks protected from sunlight.

Polymeric blends were obtained by combining NRL with polyvinyl alcohol (PVA) solutions in the absence and presence of AgNPs. NRL was mixed with PVA (8%) in a flask at 8:2 (*W*/*W*). This mixture was stirred at room temperature for 15 min at 800 RPM. Then, 3% (*W*/*V* of Polymeric blend) of AgNPs was added to the mix.

The solution preparations previously mentioned were all electrospun at 16 °C temperature with 54% humidity in a semi-industrial electrospinning/spraying machine (INOVENSO NanoSpinner PE-300, Başakşehir, Istanbul). The needle target distance (NT) was set to 220 cm using a high voltage supply of 28 kV. The collector was protected with aluminum foil. The syringe volume was 20 mL, and the feed rate was set to 0.1 mL/h. The needle received a positive charge from the high voltage, while the collector’s aluminum foil received a negative charge.

### 2.7. Characterization of NRL Decorated with AgNPs

Three different procedures were used to confirm the integration of AgNPs with NRL for characterization:

- Fourier transform infrared spectroscopy (FTIR) (FTIR-Bruker Alpha II) was used to analyze the chemical structure of NRL coated with AgNPs by examining their functional groups in the FTIR spectrum from 4000 to 500 cm⁻^1^.
- For Thermogravimetric Analysis (TGA), about 5 mg of nanofibers were utilized and placed in the oven of a Perkin Elmer Simultaneous Thermal Analyzer, model STA 6000. The sample was heated from 35 °C to 600 °C at a heating rate of 5 °C/min in a nitrogen environment with a 20 mL/min flow rate.
- The SEM photos and elemental analysis were conducted using the scanning electron imaging (SEM) model Scios 2 Thermo Scientific with EDAX Energy-Dispersive Spectroscopy (EDS) for high-resolution imaging.

### 2.8. Antimicrobial Activity of NRL Decorated with AgNPs

To evaluate the independent and synergistic antibacterial effects of NRL and NRL decorated with AgNPs, this experiment investigates their activity against pathogenic *K*. *pneumoniae* and *Bacillus* sp. Since electrospun nanofibers were partially solubilized during disc tests due to thickness and humidity, the analysis was adapted to directly utilize the electrospun polymeric solution. Serial dilutions were prepared for both NRL (80–0.5% *v*/*v*) and NRL + AgNPs (1000–31.25 µg/mL) within this solution to assess the minimum inhibitory concentration (MIC). Chloramphenicol was used as a positive control at 2 mg/mL. Bacterial cultures were standardized to 1 × 10^8^ CFU/mL (0.5 ± 0.033 McFarland). In a 96-well microplate, 20 µL of inoculum and 80 µL of broth were combined with 100 µL of each treatment (NRL or NRL + AgNPs) in each well. Plates were incubated for 24 h at 37 °C. Following incubation, 130 µL of resazurin (0.1%) was added to assess bacterial growth through absorbance measurement at 630 nm (BIOTEK, Synergy HTX). This design allows us to independently determine the MIC of both NRL and NRL + AgNPs, revealing the antibacterial effect of NRL itself and its potential enhancement with AgNPs, using a method compatible with the electrospun polymeric solution [25].

## 3. Results and Discussion

### 3.1. Preparation of Pomegranate Extract and Characterization

Our analysis of pomegranate peel alcoholic extract, summarized in Table 2, revealed several noteworthy findings. While the total sugar content was lower than that of the edible fruit portion (as documented by the U.S. Department of Agriculture, 2018 [26]), the extract exhibited a remarkably high antioxidant capacity of 22.44 g AA/100 g (ascorbic acid equivalent). This potent antioxidant activity, coupled with an IC50 of 92.06 µg/mL, surpasses the findings of Abdealsiede et al., 2020 [27] and significantly outperforms the antioxidant potential of fruit pulp extracts reported by Zeghad et al., 2019 [28]. We also analyzed the pulp and peels and determined that dried peels demonstrated the most robust antioxidant properties. These results underscore the superior antioxidant properties of pomegranate peels, particularly in their dried form.

The analysis of the pomegranate peel extract yielded detailed insights into its phenolic and flavonoid content. Concerning total phenols, our results align with the findings of Reza et al., 2011 [29], who reported values ranging from 11.62 to 21.03 mg of gallic acid/g of extract, highlighting potential variations based on the specific pomegranate variety. Similarly, our results are consistent with the range of 126.11 to 212.48 mg of gallic acid/g of extract observed by Orak et al., 2012 [30] across four pomegranate peel varieties. Factors such as cultivar and geographic origin likely contribute to these observed differences. It is worth noting that, compared to other fruits like oranges (with a reported value of 41.7 mg of gallic acid/g of dry sample), pomegranate peel maintains a high concentration of these beneficial polyphenols [31]. While the whole pomegranate fruit contains various polyphenols (including punicalagin isomers, ellagic acid derivatives, and anthocyanins), the peel boasts a particularly promising composition rich in hydrolyzable tannins such as ellagitannins, proanthocyanidins, and flavonoids [11].

Our analysis determined a total flavonoid content of 15.27 mg catechin/g of sample, within the range of 18.61 to 36.40 mg catechin/g of extract reported by Reza et al., 2011 [29]. Interestingly, Orak et al., 2012 [30] measured quercetin, finding values between 9.44 and 20.52 µg/mg. Our observed flavonoid-to-phenol ratio (0.128) supports the relationship established in the literature.

The extract’s remarkable reducing capacity (648.46 mg ascorbic acid/g sample) exceeds that of *Passiflora mollisima* (41.27 mg ascorbic acid/g sample) and *Mangifera indica* (13.7 mg ascorbic acid/g sample), highlighting the presence of compounds within the peel capable of reducing Fe^3+^ ions to Fe^2+^. This positions pomegranate peel extract as a potential agent for metal nanoparticle synthesis [32].

Pomegranate peel extract exhibits potent antioxidant capacity, surpassing that of the fruit pulp. This activity is likely attributed to the high content of phenolic compounds, particularly flavonoids, within the extract. These findings highlight its potential as a source of naturally derived antioxidants. Recent studies suggest a broader functionality for pomegranate peel extract, extending beyond its antioxidant properties, showing its efficacy as a multi-functional agent, acting as a reducing, capping, and stabilizing agent during metal nanoparticle synthesis [33,34,35]. This multi-functionality highlights its potential for developing sustainable and ecofriendly industrial processes. Further research is needed to explore the diverse functionalities and potential applications of pomegranate peel in different industries.

### 3.2. Biogenic Synthesis of AgNPs and Optimization Process

Due to a lack of clear consensus within the scientific community regarding the optimal conditions for synthesizing AgNPs on a nanometric scale, we set out to investigate factors influencing their formation [36,37]. Building upon the work of Mat Yusuf et al., 2020 [38] and others, we focused on the impact of temperature, stirring speed, silver nitrate concentration, and reaction time within the synthesis process. Therefore, we used a Design of Experiments (DOE) approach, specifically a Box–Behnken design (DBB) with 3 levels and 27 distinct experimental conditions (details in Table S1). Our primary focus was to evaluate how the selected variables impact the resulting size of the AgNPs. For this, AgNPs were synthesized using a pomegranate alcoholic extract mixed with AgNO3 (10 mM) in a 1:5 ratio and stirred continuously for 3 h. The pH was maintained at 8 for optimal production yield.

Surprisingly, within the variable ranges tested, our optimization process revealed no statistically significant effects on AgNP diameter size (Figure S1). This result and the low R-squared values indicate that the variables in the range chosen have minimal influence on nanoparticle size. Of the experimental conditions tested, we selected assay number 16 for further production. This decision was based on the promising nanoparticle size, low reaction time, and consistent 65% AgNP production yield observed upon the repetition and scaling of this process.

**Figure 1:**
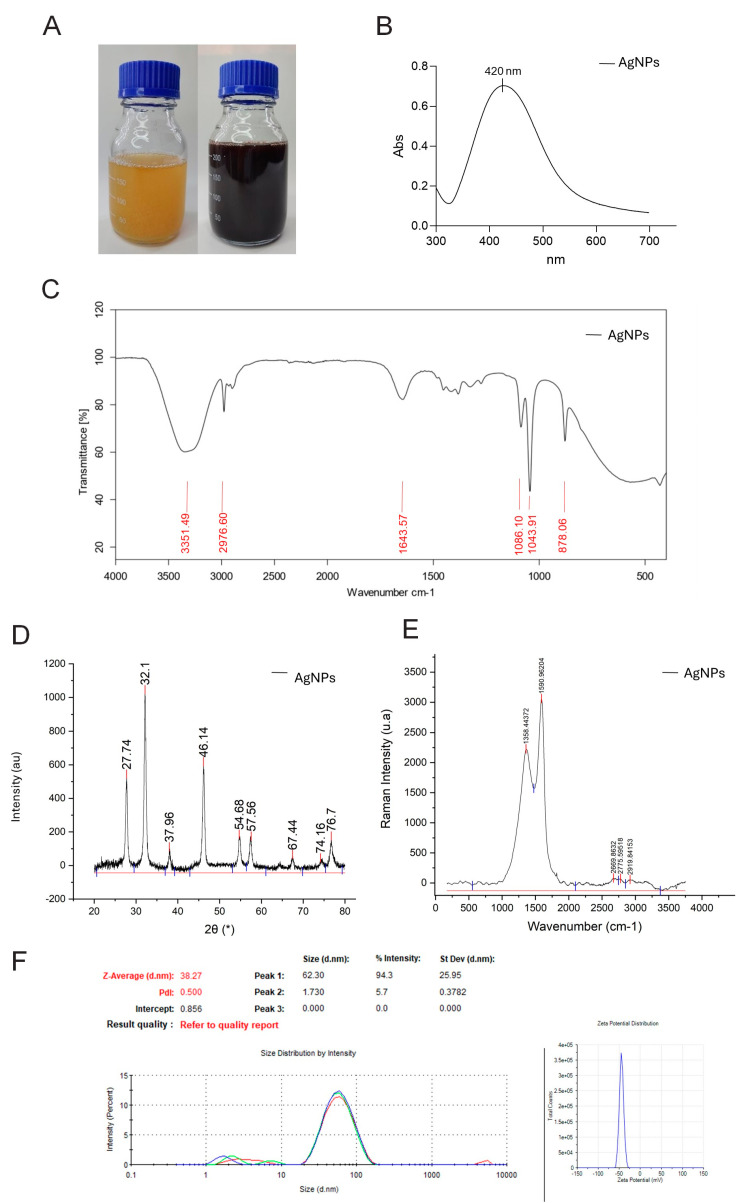
Characterization of green synthesized silver nanoparticles (AgNPs): (**A**) visual inspection of color change as silver nanoparticles synthesized; (**B**) UV-vis spectra; (**C**) FTIR spectrum analysis; (**D**) XRD analysis; (**E**) Raman analysis; (**F**) DLS and Potential Zeta analysis.

Notably, in Figure 1A, the synthesis of AgNPs using *Punica granatum* extract was visually evident by a color change from yellowish-orange to reddish-brown upon adding silver nitrate. Further adjustment of the pH to 8 led to a yellowish-black color, indicating the formation of AgNPs [39]. This observation underscores the critical role of pH in AgNP synthesis, as it directly influences size and shape [40]. Acidic environments favor smaller particles, while alkaline conditions promote larger sizes due to differences in ion density and diffusion rates [41,42]. The pH range used in our study likely did not cause significant antioxidant degradation within the *Punica granatum* extract.

### 3.3. Characterization of AgNPs

The biosynthesis of AgNPs using *Punica granatum* extract was successfully confirmed through various characterization techniques. UV-vis spectroscopy revealed a prominent absorption peak at 420 nm across all experimental designs (Figure 1B), a characteristic peak of AgNPs surface plasmon resonance [43,44].

FTIR analysis provided insights into the functional groups responsible for AgNP reduction and stabilization (Figure 1C). The peak at 3351.49 cm^−1^ suggests the involvement of -OH (alcohol) and/or N-H (amine) groups from phenolic and alcohol compounds in the extract [39,45]. These groups are often implicated in nanoparticle synthesis. Additional peaks at 2976.60 cm^−1^ (C-H stretching, carboxylic acid); 1643.57 cm^−1^ (C=O stretching, amide group); and 1086.10 cm^−1^, 1043.91, and 878.06 cm^−1^ (C-O stretching, alcohols and phenols) [46]. The peak at 1643.57 cm^−1^ was attributed to the C=O stretching of the amide group. While amides can reduce Ag^+^ to Ag^0^, their reducing power is typically weaker than polyphenols [47,48]. The presence of carboxyl, hydroxyl, and amide groups may be participating in nanoparticle synthesis [48]. Alcohols have been reported to facilitate the reduction in silver ions to AgNPs while oxidizing them to carbonyl compounds [49].

XRD analysis provided insights into the crystallinity of the synthesized AgNPs (Figure 1D). Distinct diffraction peaks at 2θ = 38, 44.28, 64.46, and 77.38, corresponding to the (111), (200), (220), and (311) lattice planes of AgNPs, respectively, were observed [39,44]. The peak at (220) indicates a face-centered cubic (fcc) crystal structure [50]. When applying the Scherrer formula, the calculated particle size was 35.64 nm, with a crystallinity of 79.43%. Similar peaks were also obtained when silver nanoparticles were synthesized using a marine alga, *Sargassum wightli* [39] and *Ocimum sanctum* leaf extract [51]. These diffraction peaks may have resulted from the capping agents that are responsible for the stabilization of nanoparticles. Similar XRD results were found in previous studies that showed the biosynthesis of AgNPs using microorganisms [39,52]. Unassigned peaks at 27.76, 32.18, and 46.18 likely correspond to phytochemicals acting as capping agents, stabilizing the nanoparticles. These findings offer details about the crystal structure, particle size, and the potential stabilizing influence of phytochemicals on the AgNPs. These findings highlight the key role of the extract’s functional groups in reducing silver ions and stabilizing the resulting nanoparticles.

The Raman spectrum (Figure 1E) reveals intense bands at 1358 and 1590 cm^−1^, indicative of the characteristic vibrational modes of silver nanoparticles. According to the authors [53,54], the band at 1369 cm^−1^ indicates the presence of silver nanoparticles. Furthermore, the spectrum exhibits bands of very low intensity at 2669 cm^−1^, corresponding to the G‘ band, as well as at 2775 and 2919 cm^−1^, potentially originating from carbonaceous materials utilized in the synthesis process [55].

Dynamic light scattering (DLS) (Figure 1F) confirmed the size distribution of our synthesized nanoparticles, revealing a range of 2 to 60 nm with an average size of 59 nm. A zeta potential of −43.2 mV (Figure 1F) indicates a negative surface charge, promoting electrostatic repulsion between particles and thus enhancing the stability of the AgNPs.

**Figure 2:**
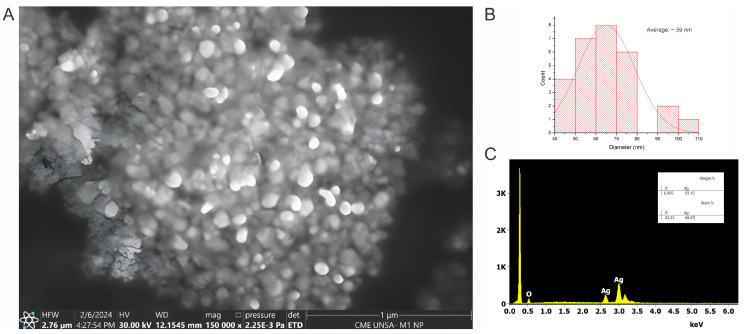
(**A**) SEM image; (**B**) histogram of the particle size; (**C**) EDS data of AgNPs.

SEM imaging (Figure 2A) shows the AgNP morphology, revealing an average particle size of 59 nm (Figure 2B). EDX analysis confirmed the elemental composition, showing a weight percentage of 93.10 for silver (Figure 2C). This provides direct visual and compositional evidence of the synthesized AgNPs.

The difference in diameter values measured by the DLS and SEM methods is naturally explained by the specificity of the techniques used [56].

These results demonstrate the successful biosynthesis of AgNPs using *Punica granatum* extract. The consistent UV-vis absorption peak and insights from FTIR, SEM, and EDX provide strong evidence for AgNP formation. The XRD pattern indicates a crystalline fcc structure and allows for particle size determination. These results align with previous studies utilizing plant extracts and microorganisms for AgNP synthesis, underscoring the potential of natural reducing agents for nanoparticle production.

### 3.4. Antimicrobial Capacity of AgNPs

**Figure 3:**
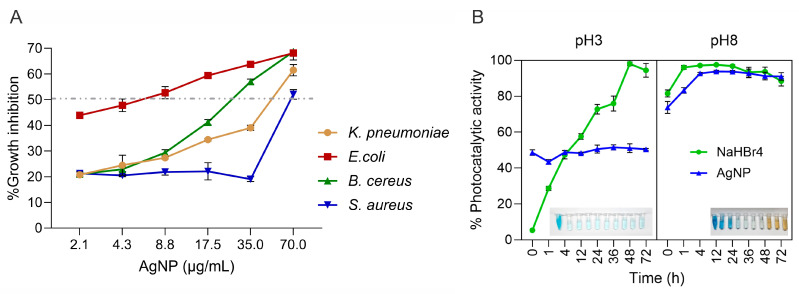
(**A**) Growth inhibition rate pathogenic bacteria based on different concentrations of AgNPs; (**B**) photocatalytic properties of AgNPs as evaluated in the MB photodegradation experiments. The IC50 value is represented by dashed lines.

We tested the antimicrobial activity of *Punica granatum*-synthesized AgNPs against a panel of microorganisms: *K. pneumoniae*, *E. coli*, *S. aureus*, and *Bacillus cereus* (Figure 3A). Our results demonstrate varying inhibitory concentrations (IC50) for each strain. Interestingly, despite the general trend in the literature favoring AgNP activity against Gram-negative bacteria, our AgNPs were highly effective against both Gram-positive (*S. aureus* and *B. cereus*, with an IC50 of 69.72 and 27.72 µg/mL, respectively) and Gram-negative species (*K. pneumoniae* and *E. coli*, with an IC50 of 51.32 and 4.87 µg/mL) (Figure S2). This finding aligns with Urnukhsaikhan et al., 2021 [57], in which synthesized using *Carduus crispus* showed effective inhibition against both bacterial types.

Additionally, our results support the findings of Ghaedi et al., 2015 [58], where AgNPs exhibited significant antibacterial activity against *E. coli*, *P. aeruginosa*, *S. aureus*, and *B. cereus*. This collective evidence underscores the broad-spectrum antimicrobial potential of green-synthesized AgNPs.

The demonstrated antimicrobial properties have implications beyond direct bacterial inhibition. Lee et al., 2003 [59] highlight the potential of AgNPs in developing antimicrobial fabrics. Their study demonstrated that fabrics treated with AgNPs retained potent activity against *S. aureus* even after repeated washing, suggesting a promising application of this technology.

Overall, our results demonstrate the effective antimicrobial activity of *Punica granatum*-derived AgNPs against a range of bacterial species. Notably, we used chloramphenicol at 2 mg/mL as our positive control, representing 100% antibacterial activity. The observed potency against Gram-negative and Gram-positive bacteria highlights the broad-spectrum potential of these nanoparticles, warranting further investigation into their use for diverse antimicrobial applications, including textile integration.

### 3.5. Photocatalytic Activity of AgNPs

Photocatalytic degradation of methylene blue (MB) using green-synthesized silver nanoparticles under solar light was successfully demonstrated, as evidenced by the initial color change of the solution. These findings align with those of Fairuzi A. et al., 2018 [60].

The photocatalytic activity was significantly influenced by the pH of the MB solution and the reaction time. At pH 3 with NaBH_4_, degradation reached 97% within 9 h. In contrast, at pH 8, degradation achieved 97% within the first hour, demonstrating a much faster reaction rate in an alkaline environment.

When using only AgNPs, degradation at pH 3 reached a plateau of 50% with no significant changes over time. However, at pH 8, degradation increased from 76% to 94% within the first 3 h of reaction, subsequently stabilizing. A later decrease in degradation percentage was observed, likely due to nanoparticle oxidation as indicated by a color change to yellowish-brown (see Figure 3B).

Alkaline conditions significantly enhance the ability of AgNPs to remove MB dye due to multiple factors. AgNPs have a negative surface charge at higher pH levels, which promotes electrostatic interaction with the cationic MB dye, increasing adsorption [61]. Additionally, the abundance of hydroxyl ions (OH-) in alkaline environments leads to the generation of highly reactive hydroxyl radicals (•OH), which act as potent oxidizing agents to degrade MB effectively. This combination of increased adsorption and enhanced oxidation power explains the superior photocatalytic degradation of MB observed in alkaline conditions [62].

Importantly, the catalytic degradation of dyes using AgNPs can be understood as an “electron shuttling” process, where AgNPs act as electron transfer facilitators—a phenomenon known as “the relay effect” [63]. While thermodynamically favorable, reducing dyes using NaBH_4_ or electron donors on the AgNPs surface (capping agents) is kinetically slow. AgNPs accelerate the degradation through their intermediate redox potential and small size, effectively catalyzing the reduction reaction [64,65].

### 3.6. Electrospinning and Characterization of NRL Decorated with AgNPs

**Figure 4:**
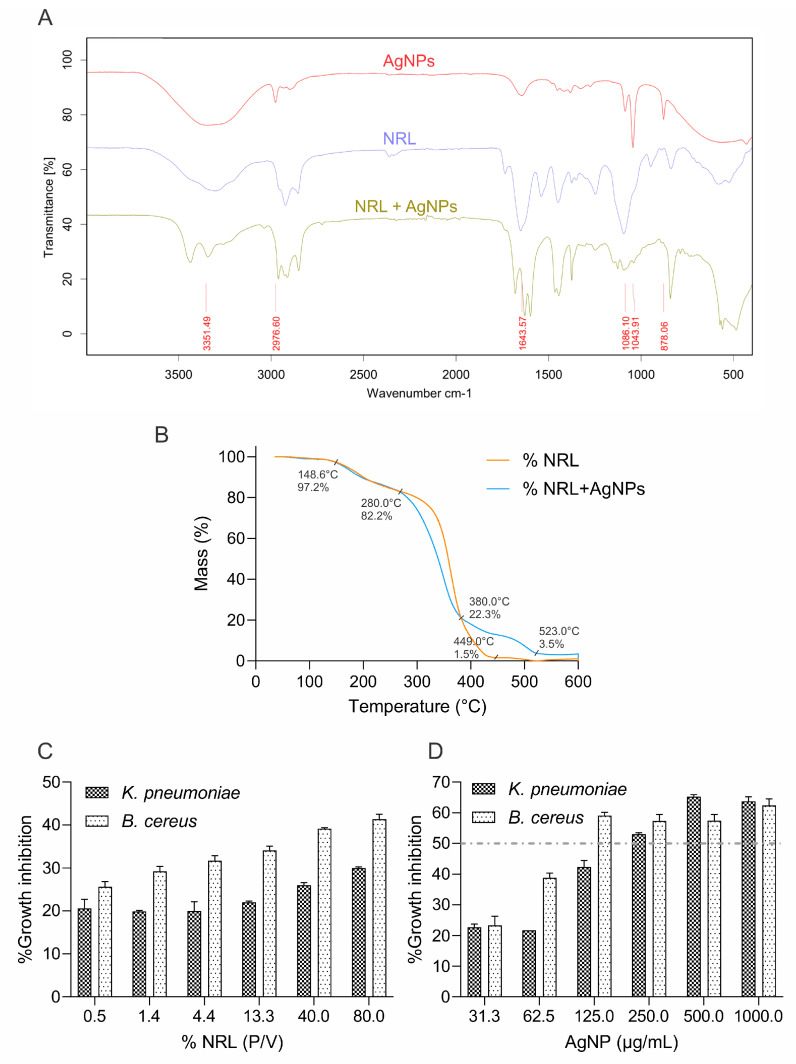
(**A**) FTIR spectra of AgNPs, NRL, and NRL + AgNPs; (**B**) thermogravimetric analysis (TGA) of NRL and NRL + AgNPs; (**C**) grow inhibition rate of NRL; (**D**) grow inhibition rate of NRL + AgNPs on pathogenic bacteria. The IC50 value is represented by dashed lines.

FTIR analysis provided insights into the chemical composition of the latex, silver nanoparticles, and their combined NRL-AgNPs system (Figure 4A). The pure latex spectrum revealed characteristic bands at 3304.26 cm^−1^ (-OH groups), 2922.49 cm^−1^ (-CH alkyl groups), 1651.07 cm^−1^ (carbonyl or C=C bonds), and 1246.95 cm^−1^ (ether or alcohol groups) [66,67]. These bands align with the expected chemical structure of rubber polymers.

The NRL-AgNPs spectrum exhibited major peaks at 582.36, 1092.92, 1375.07, 1597.86, and 3436.06 cm^−1^. Importantly, the characteristic silver band (500–600 cm^−1^), corresponding to metallic bond vibrations, confirms the successful incorporation of AgNPs within the NRL matrix. The broad, strong peak at 3000–3600 cm^−1^ suggests overlapping OH and NH_2_ stretching vibrations [68].

TGA was performed to assess the thermal stability of pure latex and NRL-AgNPs films (Figure 4B). Both samples showed gradual mass loss with increasing temperature, which can be attributed to the desorption of solvents within the film and the breakdown of organic components [69]. Pure latex showed a slow initial decrease (100–150 °C) followed by a significant drop (280 °C) attributed to solvent desorption and organic component decomposition, respectively. The presence of AgNPs impacted the thermal behavior. While initial solvent desorption was similar, the subsequent mass decrease (380 °C) was more pronounced, suggesting interaction between the nanoparticles and organic components. Remarkably, the behavior from 400 to 600 °C points towards increased thermal stability. The slower mass loss and eventual stabilization suggest potential cross-linking with the nanoparticles, hindering their release and enhancing stability. The analysis revealed a final silver residue of approximately 3.5% of the total sample mass.

In NRL, the slow mass loss between 100 °C and 150 °C likely corresponds to the evaporation of residual solvents and adsorbed water. A decrease to 80% at 280 °C indicates the thermal decomposition of the latex’s organic components. This is followed by an abrupt drop to 5% at 420 °C due to pronounced degradation of polymer chains. Further mass loss of up to 1.5% at 600 °C may be due to residual carbonization.

The NRL-AgNPs sample exhibited a similar initial mass loss (100–150 °C) due to solvent desorption. However, the more pronounced mass decrease to 20% at 380 °C suggests altered thermal decomposition behavior. This is likely due to interactions between AgNPs and the organic latex components, which influence the breakdown process. Interestingly, the slower mass decrease (400–500 °C) followed by stabilization at 10% (500–600 °C) indicates enhanced thermal stability with adding AgNPs. This improvement may be due to a cross-linking network within the NRL matrix and the presence of thermally stable silver nanoparticles.

**Figure 5:**
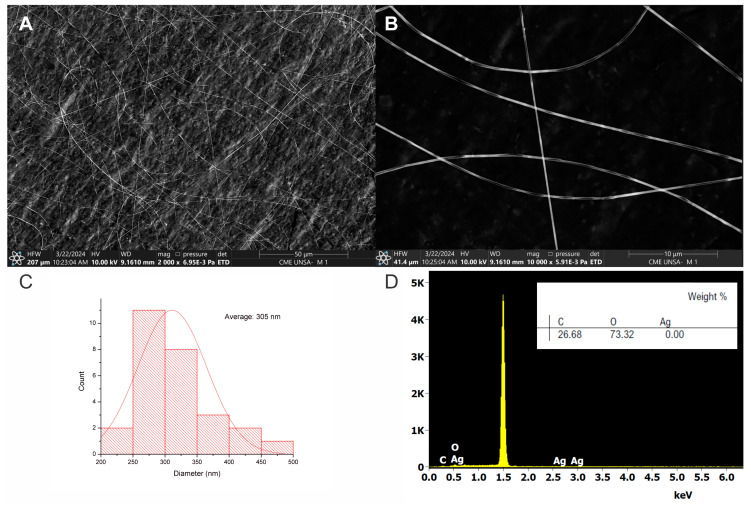
SEM images of pure NRL nanofibers: (**A**) overview at 50 µm scale; (**B**) detail at 10 µm scale showing smooth fibers; (**C**) fiber diameter distribution histogram (average diameter = 305 nm); (**D**) EDS spectrum confirming the composition of NRL.

SEM analysis revealed the successful fabrication of uniform NRL nanofibers with an average diameter of 305 nm (Figure 5C). The fibers exhibited a smooth surface and were defect-free, as seen in Figure 5A,B. This uniformity highlights the effectiveness of the fabrication process. It suggests predictable properties and controlled interactions, making these nanofibers promising for applications where surface area, pore size, and consistency are crucial. Additionally, EDS analysis confirmed the absence of silver (Figure 5D), further validating the successful synthesis of the NRL nanofibers.

**Figure 6:**
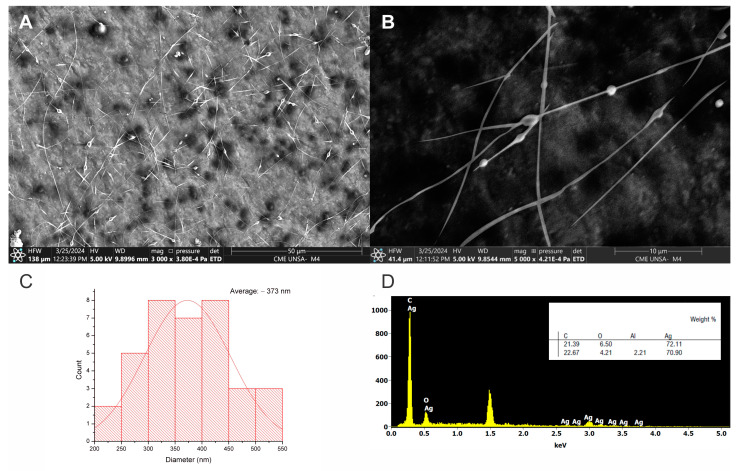
SEM images of NRL + AgNPs nanofibers: (**A**) overview at 50 µm scale; (**B**) detail at 10 µm scale showing AgNPs agglomerations; (**C**) fiber diameter distribution histogram (average diameter = 373 nm); (**D**) EDS spectrum confirming AgNPs presence.

SEM analysis of NRL + AgNPs nanofibers with an average diameter of 373 nm (Figure 6C). While some agglomeration of AgNPs was observed (Figure 6A,B), potentially due to the viscosity of the polymeric solution or interactions with NRL components such as proteins, the overall fiber morphology remained consistent. These biomolecules in NRL can interact with the AgNP surface, acting as bridges between nanoparticles, altering their surface charge, or interfering with stabilizing agents. Additionally, NRL’s pH and ionic strength can further influence AgNP stability; variations in pH might affect nanoparticle surface charge and protein behavior, while high ionic strength can reduce repulsive forces between AgNPs.

The presence of AgNPs was confirmed by EDS analysis, showing a 72% weight content in the fiber analyzed (Figure 6D). Incorporating AgNPs suggests a potential for enhanced properties such as antimicrobial activity or conductivity. However, the observed agglomeration may impact the distribution of the AgNPs within the nanofibers. Potentially, by adjusting solvent ratios or incorporating surfactants, further optimization of the fabrication process could improve AgNPs dispersion and maximize the potential benefits of their inclusion.

Interestingly, the observed agglomeration of AgNPs could potentially slow their release into the environment compared to their free form, as we also observed in our parallel studies of composite formation (Figure S4). This slower release might mitigate environmental concerns, but further analysis must be performed to determine the precise liberation rate, assess the long-term stability of agglomerations, and evaluate their specific environmental impacts. These studies will clarify this phenomenon’s potential ecological safety benefits and long-term implications.

Overall, the combined FTIR, TGA, and SEM results provide evidence of the successful integration of AgNPs into the NRL matrix. The FTIR analysis confirms AgNP incorporation, while TGA reveals the significant impact of AgNPs on the thermal behavior of NRL, notably improving its thermal stability. These findings have important implications for the stability and potential applications of NRL-AgNPs composite films.

### 3.7. Antimicrobial Activity of NRL Decorated with AgNPs

The initial approach for characterization involved cutting the electrospun nanofibers into discs for analysis on agar plates. However, due to the inherent thickness and humidity of the material, the discs exhibited uneven shapes and displayed signs of partial solubilization. The analysis strategy was adapted to address this limitation and ensure accurate characterization. Instead of using fiber discs, the electrospun polymeric solution was directly used for analysis.

The assay evaluated the antibacterial efficacy of NRL alone and with AgNPs against *K. pneumoniae* and *B. subtilis*. No antibacterial effect was observed against *S. aureus* and *E. coli*. The results demonstrate a gradual increase in the antibacterial effect of NRL alone, from 19% to 30%, as its concentration rises from 0.49% to 80% (Figure 4C).

This indicates inherent antibacterial properties within the NRL, probably due to its content in proteins and molecules, such as hevein-like proteins, lysozyme-like enzymes, chitinases, and small peptides, which can disrupt microbial cell structures and processes that target fungal cell walls. Additionally, NRL’s slightly acidic pH and the physical barrier formed by its dried film can impede microbial growth [70,71].

Adding AgNPs (31–1000 µg/mL) significantly enhances antibacterial activity across all NRL concentrations evaluated (Figure 4D). A synergistic effect between NRL and AgNPs is evident, with inhibition increasing from 23% to 63%. This enhancement is likely due to AgNPs’ multiple modes of action, including their ability to penetrate bacterial cells, disrupt membranes, and generate reactive oxygen species that cause cellular damage.

The optimal MIC concentration of AgNPs was selected at 250 µg/mL since approximately 50% inhibition was observed. However, a saturation effect is evident beyond a certain AgNP concentration, particularly at higher NRL levels. This suggests a limit to the achievable synergistic effect. These consistent trends underscore the reliability of the experimental setup. In the case of *Bacillus cereus*, the antibacterial effect of NRL alone ranged from 26% to 42%, while adding AgNPs resulted in 25% to 63% inhibition. The MIC was selected at 125 µg/mL (Figure S3).

These findings suggest the potential of NRL-AgNPs composites as effective antibacterial agents against *K. pneumoniae* and *B. cereus*. Further investigation is warranted to understand the underlying mechanisms and potential applications in antimicrobial settings. Exploring their cytotoxicity and biocompatibility for safe utilization in medical and industrial contexts is also crucial.

## 4. Conclusions

The present study successfully demonstrates the biosynthesis of AgNPs using *Punica granatum* extract. Characterization techniques (UV-vis, FTIR, RAMAN, TGA, SEM, EDX, and XRD) confirmed the formation of spherical, crystalline AgNPs with an average diameter of 59 nm. The study highlights the key role of functional groups within the extract in reducing silver ions and stabilizing the nanoparticles.

The AgNPs exhibited significant photocatalytic activity in degrading methylene blue, especially under alkaline conditions. This supports the “electron shuttling” mechanism and suggests broad potential for environmental remediation applications. Moreover, the green-synthesized AgNPs demonstrated activity against *K. pneumoniae*, *E. coli*, *B. cereus*, and *S. aureus*, underscoring their broad-spectrum antimicrobial capabilities.

Moreover, incorporating AgNPs into NRL matrices sourced from eco-friendly Peruvian Amazonian communities significantly enhanced thermal stability and antimicrobial properties. The NRL/AgNPs nanofibers, with diameters ranging from 300 to 373 nm, showed activity against *K. pneumoniae* and *B. cereus*. This makes AgNPs-impregnated NRL a promising candidate for applications requiring sustainability and antimicrobial functionality, such as in developing smart textiles, biomedical textiles, and filtration materials.

This work demonstrates the use of *Punica granatum* extract as a versatile, eco-friendly, cost-effective reducing and capping agent for AgNP synthesis. By utilizing pomegranate waste, this study addresses an environmental challenge in Arequipa. It reinforces the value of exploring sustainable nanoparticle production sources and highlights Peruvian NRL’s potential. Future research should optimize AgNP synthesis using this method, explore their incorporation into other eco-friendly matrices, and rigorously evaluate their performance in specific applications.

## Figures and Tables

**Table 1:** Variables and their coded levels, Box–Behnken design for 3 factors.

		Levels		
Independent variable	Unit	−1	0	+1
X1: Temperature	°C	20	42.5	65
X2: Agitation	RPM	300	450	600
X3: AgNO_3_ concentration	mM	1	5.5	10
X4: Reaction time	h	1	3	5

**Table 2:** Summary of *Punica granatum* peel hydroalcoholic extract characterization.

	Total Sugars mg Glucose/g Sample	Antioxidant Capacity mg AA/100 g Sample	Total Phenols mg AG/g Sample	Total Flavonoids mg Cat/g Sample	Reducing Capacity mg AA/g Sample
Value	269.12	22,444.03	89.84	15.27	648.46
Standard deviation	0.0012	0.9976	0.0164	0.0207	0.0194

## Data Availability

The original contributions presented in the study are included in the article/Supplementary Materials, further inquiries can be directed to the corresponding author/s.

## Supplementary Materials

The following supporting information can be downloaded at: https://www.mdpi.com/article/10.3390/polym16111531/s1, Table S1. Box-Behnken design of experiments (DoE) investigates the relationship between synthesis parameters and AgNPs diameter. Figure S1. Graph of ANOVA analysis and predicted values vs. observed values from software STATISTICA^®^. Figure S2. Schematic of the 96-well resazurin broth microdilution assay. Blue wells indicate inhibited bacterial growth, while pink wells represent active organisms. Intensity variations in lanes 1–6 (left to right) reflect the effects of increasing AgNPs concentrations. Lanes 7 and 8 are positive and negative controls, respectively. Test performed in quintuplicate. Figure S3. A schematic of the 96-well resazurin broth microdilution model. Wells with blue color indicate inhibited growth; pink wells indicate active organisms. Variations in blue/pink shades between lanes 1–9 are due to the effect of different NRL and NRL+ AgNP concentrations. Figure S4. SEM images of NRL + AgNPs composite. and EDS spectrum confirming AgNP presence.
