# Supplementary material for: Green Synthesis of Silver Nanoparticles Mediated by Punica granatum Peel Waste: An Effective Additive for Natural Rubber Latex Nanofibers Enhancement
Source: Polymers (Basel). 2024 May 29;16(11):1531. doi: 10.3390/polym16111531 (PMC11174564; doi:10.3390/polym16111531)
Supplement: Supplementary file 1 [file polymers-16-01531-s001.zip › polymers-2983186-supplementary.pdf]

Supplementary material

Green Synthesis of Silver Nanoparticles Mediated by *Punica granatum* Peel Waste: An Effective Additive for Latex Nanofiber Enhancement

Talia S. Echegaray-Ugarte[1], Andrea L. Cespedes-Loayza[1], Jacqueline L. Cruz-Loayza[1], Luis A. Huayapa-Yucra, Isemar Cruz, Júlio Cesar de Carvalho[2] , and Luis Daniel Goyzueta-Mamani[3*]

Table S1. Box-Behnken design of experiments (DoE) investigates the relationship between synthesis parameters and AgNPs diameter.

Sample	Synthesis conditions				UV-VISIBLE		Particle size (ZETASIZER)	
	Temperature (ºC)	Agitation (rpm)	$AgNO_3$ Concentration (mM)	Reaction time (h)	Maximum Absorbance	Wavelenght (nm)	Particle size (nm)	Stand. deviation
1	20	300	5.5	3	0.2684	403.0	113.17	6.05
2	65	300	5.5	3	0.3421	411.0	26.53	0.79
3	20	600	5.5	3	0.2799	400.0	251.63	1.70
4	65	600	5.5	3	0.0700	417.5	31.67	1.41
5	42.5	450	1.0	1	0.6366	411.5	11.29	0.10
6	42.5	450	10	1	0.6419	411.0	28.59	1.02
7	42.5	450	1.0	5	0.2395	403.0	20.55	1.11
8	42.5	450	10	5	0.4552	408.0	11.74	0.11
9	42.5	450	5.5	3	0.5112	407.0	42.58	0.77
10	20	450	5.5	1	0.3364	403.0	48.92	1.21
11	65	450	5.5	1	1.5212	413.0	31.10	0.62
12	20	450	5.5	5	0.4699	403.5	47.64	0.39
13	65	450	5.5	5	0.3063	408.0	26.92	0.08
14	42.5	300	1.0	3	0.3591	408.0	13.26	0.03
15	42.5	600	1.0	3	0.7739	411.0	11.11	0.03
16	42.5	300	10	3	1.2589	413.0	14.37	0.09
17	42.5	600	10	3	0.4069	409.0	44.11	0.97
18	42.5	450	5.5	3	0.7557	408.0	31.95	0.15
19	20	450	1.0	3	0.6625	410.0	11.65	0.09
20	65	450	1.0	3	0.3573	405.0	21.34	1.24
21	20	450	10	3	0.3029	411.0	21.89	3.50

22	65	450	10	3	1.2503	416.0	28.23	1.35
23	42.5	300	5.5	1	0.5047	402.0	25.78	0.04
24	42.5	600	5.5	1	0.7793	406.5	31.95	0.24
25	42.5	300	5.5	5	0.4271	403.0	39.55	0.18
26	42.5	600	5.5	5	0.8484	415.0	26.81	0.07
27	42.5	450	5.5	3	0.2309	405.0	121.93	2.63

ANOVA; Var.:Size; R-sqr=.4646; Adj:.22665 (Spreadsheet3)
4 3-level factors, 1 Blocks, 27 Runs; MS Residual=1899.513
DV: Size

Factor	SS	df	MS	F	p
(1)Temperature(L)	9026.12	1	9026.116	4.751804	0.042789
Temperature(Q)	4899.45	1	4899.446	2.579317	0.125669
(2)Metal Concentration(L)	2258.31	1	2258.312	1.188890	0.289936
Metal Concentration(Q)	78.97	1	78.973	0.041575	0.840719
(3)Agitation(L)	297.31	1	297.306	0.156517	0.697033
Agitation(Q)	9747.78	1	9747.778	5.131724	0.036066
(4)Agitation time(L)	1.63	1	1.628	0.000857	0.976967
Agitation time(Q)	2956.20	1	2956.199	1.556293	0.228196
Error	34191.24	18	1899.513		
Total SS	63861.37	26			

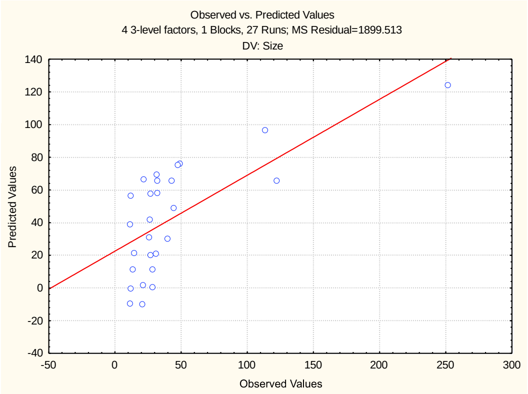

Figure S1. Graph of ANOVA analysis and predicted values vs. observed values from software STATISTICA ®.

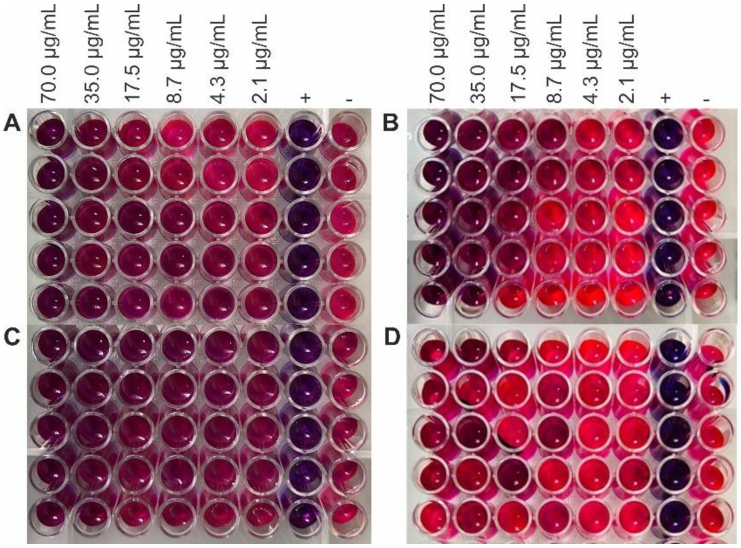

Figure S2. Schematic of the 96-well resazurin broth microdilution assay. Blue wells indicate inhibited bacterial growth, while pink wells represent active organisms. Intensity variations in lanes 1-6 (left to right) reflect the effects of increasing AgNPs concentrations. Lanes 7 and 8 are positive and negative controls, respectively. Test performed in quintuplicate.

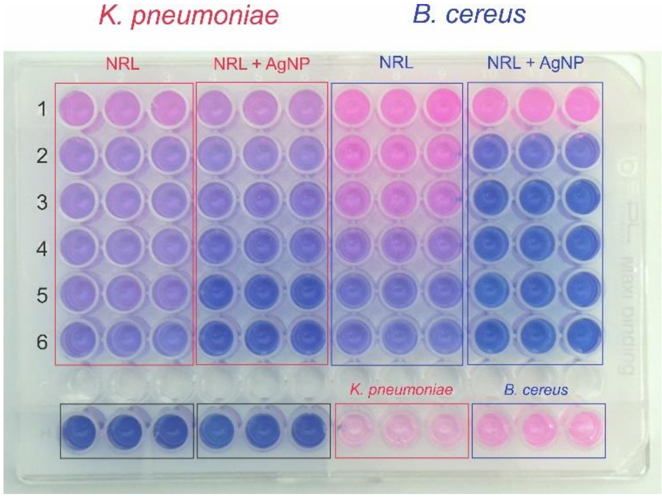

Figure S3. A schematic of the 96-well resazurin broth microdilution model. Wells with blue color indicate inhibited growth; pink wells indicate active organisms. Variations in blue/pink shades between lanes 1-9 are due to the effect of different NRL and NRL+ AgNP concentrations.

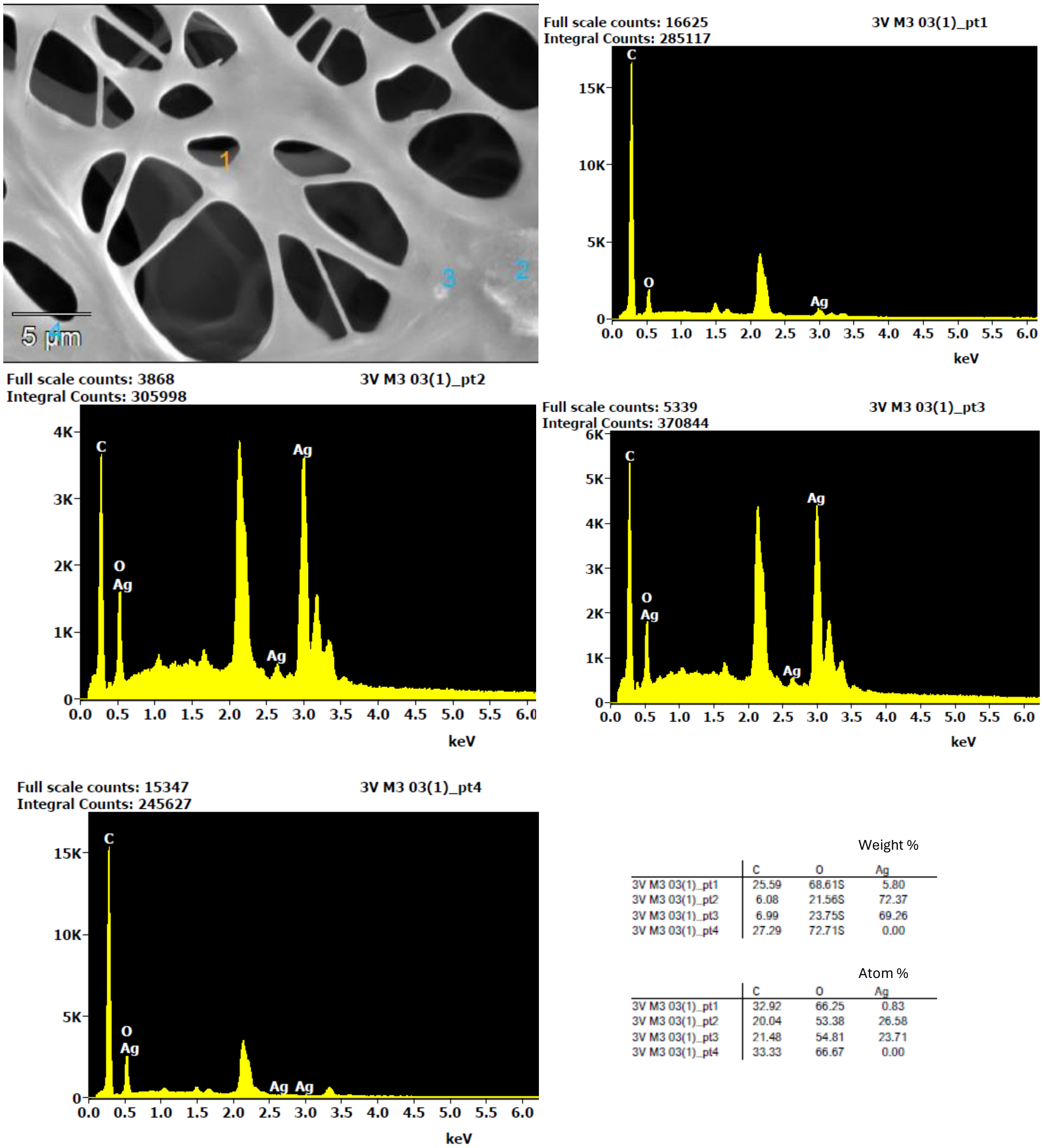

Weight %

	C	O	Ag
3V M3 03(1)_pt1	25.59	68.61S	5.80
3V M3 03(1)_pt2	6.08	21.56S	72.37
3V M3 03(1)_pt3	6.99	23.75S	69.26
3V M3 03(1)_pt4	27.29	72.71S	0.00

Atom %

	C	O	Ag
3V M3 03(1)_pt1	32.92	66.25	0.83
3V M3 03(1)_pt2	20.04	53.38	26.58
3V M3 03(1)_pt3	21.48	54.81	23.71
3V M3 03(1)_pt4	33.33	66.67	0.00

Figure S4. SEM images of NRL + AgNPs composite. and EDS spectrum confirming AgNP presence.
